# Refeeding syndrome and vitamin B1 deficiency in a young man with normal body mass index following starvation in the COVID‐19 era

**DOI:** 10.1002/jgf2.729

**Published:** 2024-10-21

**Authors:** Naoaki Tsuji, Hisatoshi Okumura, Satoshi Inaba, Akihito Kaneko, Atsushi Kawashima

**Affiliations:** ^1^ Department of Respiratory Medicine Otsu City Hospital Shiga Japan; ^2^ Department of Emergency Medicine Kyoto Prefectural University of Medicine Kyoto Japan; ^3^ Division of General Internal Medicine Fukuchiyama City Hospital Kyoto Japan; ^4^ Department of Pathology Kyoto Prefectural University of Medicine Kyoto Japan

**Keywords:** COVID‐19, refeeding syndrome, vitamin B1 deficiency, Wernicke encephalopathy

## Abstract

COVID‐19 has spread worldwide and significantly influenced economies. Refeeding syndrome (RFS) is a potentially fatal abnormalities of electrolytes and fluid that can occur in malnourished patients undergoing mechanical refeeding. Herein, we report the case of a man in his 20s with a normal body mass index who presented with RFS and vitamin B1 deficiency. Although it was uncommon under normal circumstances, it occurred because of the severe social situations that were prevalent in the COVID‐19 era. In this era, physicians should carefully evaluate their patients' nutritional status to identify those at risk for RFS, even in young individuals.

## 1 BACKGROUND

COVID‐19 endemic is still significantly influencing national economies. The ensuing unemployment is substantial issue. In Japan, it was reported that the risk of unemployment was highest among young people in the COVID‐19 era.^1^

Refeeding syndrome (RFS) is commonly defined as a potentially fatal change in fluid and electrolyte levels that can occur in malnourished patients undergoing mechanical refeeding.^2^

Herein, we report the case of a man in his 20s with normal body mass index (BMI) who presented with RFS and vitamin B1 deficiency. Although it was uncommon under normal circumstances, it occurred because of the severe social situations that were prevalent in the COVID‐19 era.

## 2 CASE PRESENTATION

A Japanese man in his 20s was brought to the emergency department with convulsions. Later, we obtained a more detailed history from the mother, incredulous history of not taking enough nutrition for a month before the onset of convulsion.

On examination, he had a blood pressure of 140/82 mmHg, a pulse rate of 158 beats per minute, a respiratory rate of 20 breaths per minute, a temperature of 35.6°C, and an oxygen saturation of 98% on ambient air. The Glasgow Coma Scale score was 14 (E3, V5, and M6). His height was 175.5 cm, and his body weight was 71.1 kg, with a BMI of 23.1. Physical examinations, including neurological ones, were within the normal range.

Laboratory values were as follows: leukocyte count, 10,150/μL (84% neutrophils); blood glucose, 209 mg/dL; serum sodium, 135 mEq/L; serum potassium, 2.7 mEq/L (Table 1). A computed tomography scan of head did not show any abnormalities. Moreover, his spinal fluid test showed normal results.

**TABLE 1:** Laboratory data.

Variables	Reference range, Adults	On presentation	Two days before presentation
Leukocyte count (per μL)	3600–9000	10,150	8400
Hemoglobin (g/dL)	11.3–15.2	16.9	18.8
Mean corpuscular volume (fL)	83.0–100.0	88.8	89.0
Platelet count (per μL)	12.0–34.0 × 10^4^	25.8 × 10^4^	29.9 × 10^4^
Blood glucose level (mg/dL)	70–110	209	164
Aspartate aminotransferase (U/L)	8–38	26	26
Alanine aminotransferase (U/L)	4–44	16	12
Lactate dehydrogenase (U/L)	124–222	289	233
Creatine kinase (U/L)	56–244	148	105
Creatinine (mg/dL)	0.30–0.80	0.78	0.72
C‐reactive protein (mg/dL)	<0.3	3.32	0.23
Serum sodium (mEq/L)	136–147	135	147
Serum potassium (meq/L)	3.6–5.0	2.7	4.0
Serum chloride (meq/L)	98–109	85	95
Serum calcium (mg/dL)	8.7–10.1	10.0	—
Serum magnesium (mg/dL)	1.8–2.6	2.3	3.1
Serum phosphorus (mg/dL)	2.4–4.3	1.6	4.2
Vitamin B1 (μg/dL)	24–66	19.3	23

We obtained details on his additional medical history from his mother. He had no choice but to take a day job after the company he had been offered to work for canceled his job offer. Subsequently, he quit the day labor job and fell into financial hardship. According to him and his mother, which was difficult for us to believe, he survived the 40 days prior to his visit by consuming only the leftover food in his refrigerator. His initial body weight was nearly 90 kg (BMI: 29 kg/m^2^). Two days before his presentation, he was found lying outdoors and transferred to another hospital. In that time, he was diagnosed with dehydration and was discharged home after receiving fluids. Then, he ate an oyakodon, prepared by his mother. One day before his presentation, his mother took him home to her house out of concern. On the day of his presentation, he had tonic–clonic convulsions twice with loss of consciousness in the morning. He had no remarkable medical history or medication use. He was neither a smoker nor a drinker of alcohol.

**FIGURE 1:**
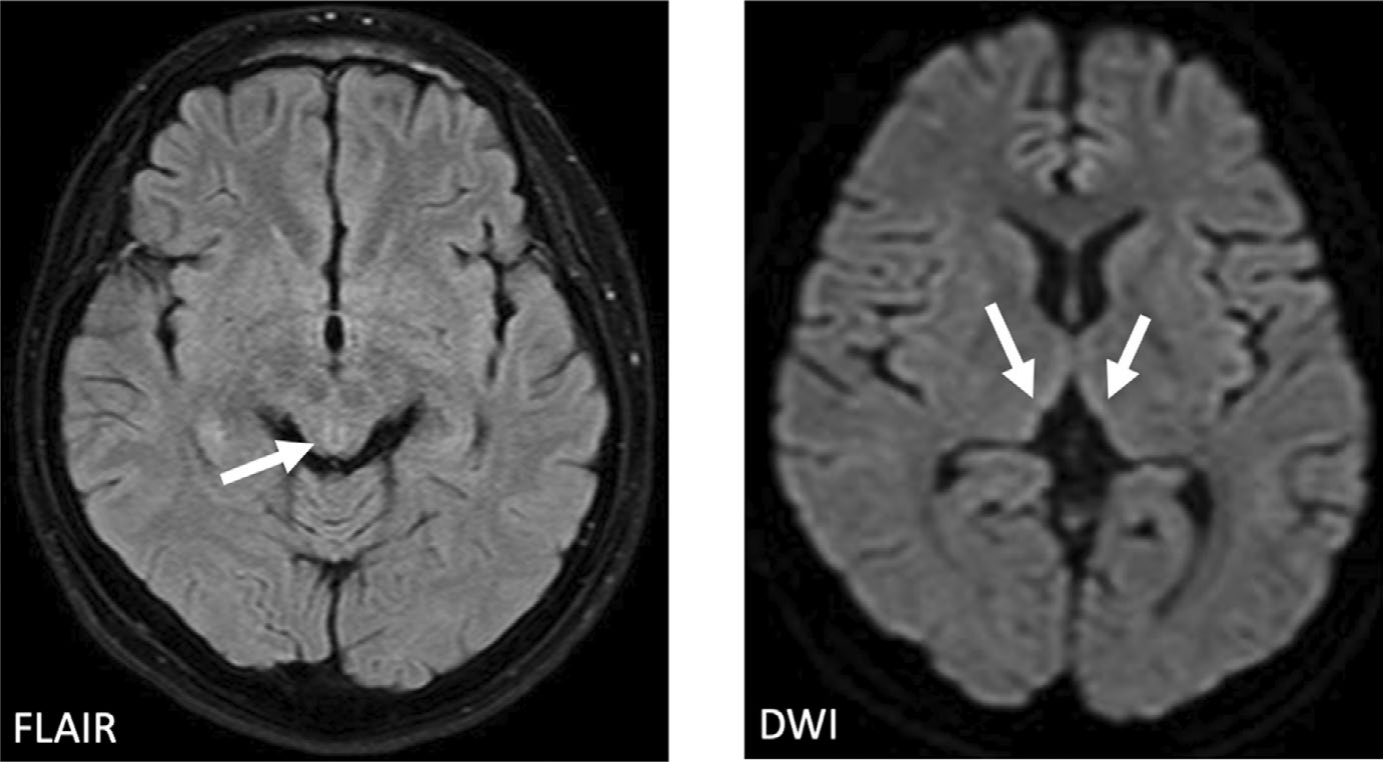
Magnetic resonance imaging (MRI) of the patient's brain. Diffusion‐weighted image (DWI) and fluid attenuated inversion recovery (FLAIR) revealed increased signal intensity in the bilateral dorsomedial thalami and around the cerebral aqueduct (white arrows).

From the first day of admission, intravenous thiamine was administered. The following laboratory results taken at the emergency department were revealed: serum magnesium, 2.3 mg/dL; serum phosphorus, 1.6 mg/dL; vitamin B1, 19.3 μg/dL (Table 1). During his admission, he complained of double vision, and ataxic gait were evident. Magnetic resonance imaging of the brain revealed increased signal intensity in the bilateral dorsomedial thalami (Figure 1), which was consisted with Wernicke encephalopathy. His ataxia and nystagmus became mild with long‐term administration of high‐dose vitamin B1.

There was an unusual situation in which he did not ask his mother for financial assistance, even though he was in such financial difficulties that he could not eat a meal for 40 days. Although he was referred to a psychiatrist, no psychological illness, including developmental disorder, was pointed out. On the 26th day after admission, he was discharged home. We introduced him and his family to the welfare services at the city office. He is currently doing well.

## 3 DISCUSSION

COVID‐19 has spread worldwide and has had a significant impact on the global economy. Because of COVID‐19, people were exposed to the greatest loss of labor opportunities worldwide.^3^ In Japan, young workers were more likely to be unemployed than workers in the middle or older age group.^1^, ^4^

Physicians should carefully assess the nutritional status of patients to detect patients at risk of RFS and vitamin B1 deficiency, and provide appropriate nutrient therapy. In most cases, RFS has been reported to occur within the first 3 days of the start of nutritional support.^5^ Hypophosphatemia has been identified as the hallmark biochemical marker of RFS.^2^ Hypomagnesaemia and thiamine deficiency may occur.^6^ The risk factors include a low BMI (especially BMI < 16), anorexia nervosa, chronic alcoholism, oncology patients, older patients, little or no nutritional intake for >10 days, unintentional weight loss (especially more than 15% in the past 3–6 months), and long‐term use of antacids and diuretics.^2^ Malnutrition threatens up to 50% of patients admitted to acute psychiatric units, especially in self‐neglect, alcohol and drug dependency, depression, schizophrenia, and dementia.^7^, ^8^ Physicians should be aware of the malnourished patients who are at risk of RFS, and accordingly, measure the serum magnesium, phosphorus, and vitamin B1 levels. The red herring of this case was that his BMI was within the normal range, and he did not appear to be starved or at risk of RFS. When estimating the risk of RFS, it is important to consider not only considerably thin patient but also the history of weight loss.

Refeeding should be started at a low‐energy replacement.^9^ Monitoring clinical and biological parameters is essential in managing patients at high risk of RFS. In the emergency department, if the risk for RFS and vitamin B1 deficiency are not identified before starting calories, RFS and Wernicke encephalopathy may occur.^10^ Decisions about thiamine supplementation should rely on a clinical judgment of the risk for vitamin B1 deficiency rather than awaiting the result of the serum thiamine test.

In conclusion, physicians should assess the nutritional status of patients to detect those at risk of RFS and vitamin B1 deficiency and provide appropriate nutrient therapy in the COVID‐19 era. Our experience should be documented as a case caused by the difficult social situations because of COVID‐19.

## CONFLICT OF INTEREST STATEMENT

The authors have stated explicitly that there are no conflicts of interest in connection with this article.

## ETHICS STATEMENT

Ethics approval statement: None.

Patient consent statement: The authors have obtained patient consent.

Clinical trial registration: None.

## References

[1] Kuroishi M , Nagata T , Hino A , Tateishi S , Ogami A , Tsuji M , et al. Prospective cohort study of sociodemographic and work‐related factors and subsequent unemployment under COVID‐19 pandemic. Int J Environ Res Public Health. 2022;19:6924. 35682507 10.3390/ijerph19116924 PMC9180179

[2] Mehanna HM , Moledina J , Travis J . Refeeding syndrome: what it is, and how to prevent and treat it. BMJ. 2008;336:1495–1498. 18583681 10.1136/bmj.a301 PMC2440847

[3] Kawohl W , Nordt C . COVID‐19, unemployment, and suicide. Lancet Psychiatry. 2020;7:389–390. 32353269 10.1016/S2215-0366(20)30141-3 PMC7185950

[4] Fukai T , Ichimura H , Kawata K . Describing the impacts of COVID‐19 on the labor market in Japan until June 2020. Jpn Econ Rev. 2021;72:439–470. 10.1007/s42973-021-00081-z PMC8286989 34305434

[5] Friedli N , Stanga Z , Culkin A , Crook M , Laviano A , Sobotka L , et al. Management and prevention of refeeding syndrome in medical inpatients: an evidence‐based and consensus‐supported algorithm. Nutrition. 2018;47:13–20. 29429529 10.1016/j.nut.2017.09.007

[6] Maiorana A , Vergine G , Coletti V , Luciani M , Rizzo C , Emma F , et al. Acute thiamine deficiency and refeeding syndrome: similar findings but different pathogenesis. Nutrition. 2014;30:948–952. 24985016 10.1016/j.nut.2014.02.019

[7] Abayomi J , Hackett A . Assessment of malnutrition in mental health clients: nurses' judgement vs. a nutrition risk tool. J Adv Nurs. 2004;45:430–437. 14756837 10.1046/j.1365-2648.2003.02926.x

[8] Gray GE , Gray LK . Nutritional aspects of psychiatric disorders. J Am Diet Assoc. 1989;89:1492–1498. 2677098

[9] De Silva A , Nightingale JMD . Refeeding syndrome: physiological background and practical management. Frontline Gastroenterol. 2020;11:404–409. 32884632 10.1136/flgastro-2018-101065 PMC7447285

[10] da Silva JSV , Seres DS , Sabino K , Adams SC , Berdahl GJ , Citty SW , et al. ASPEN consensus recommendations for refeeding syndrome. Nutr Clin Pract. 2020;35:178–195. 32115791 10.1002/ncp.10474

